# What Do You Charge?

**Published:** 1876-10

**Authors:** Thos. K. Beecher


### WHAT DO YOU CHARGE?

THOS. K. BEECHER.

A very common question this, a question that is asked and answered every day, almost without thought, and yet sometimes one of the most difficult questions that can be asked.

The coarse commercial answer is: "Charge all that you can expect to collect; the value of anything, and therefore its proper price, is what it will fetch in open market."

As to staple articles, such as wheat, flour, butter, sugar, meats, cottons, woolens, lands, houses and such, this coarse answer, in times of general thrift, may perhaps be the best that can be made. The market price will be regulated by what we call the law of demand and supply.

As to "fancy values," also, as diamonds, trotting horses, laces, pictures, statuary, poems, lectures and sermons—articles which no man needs, yet many men long for—what to charge for these is perhaps well answered:—Charge all that you can collect.

But again and again in some of the most important and delicate relations of life the question is asked: What do you charge? Surgeons and physicians meet this question daily. Here is a wife and mother sure to die within six months except an operation be performed. "What do you charge, Doctor?" Here is a great wrong about to be perpetrated on a defenceless widow by her creditor. What do you charge, lawyer, for your counsel? Here is a capsized sail-boat and ten men and women struggling in the water. What will you charge, boatman, to pick them up? Here is Chicago on fire. What will you charge, drayman, for the use of your van six hours? In short, men often find themselves in such extremity that they are helpless. They have no chance to choose—"*Skin for skin, all that a man hath will he give for his life.*"

In all such cases it is plain that the coarse commercial rule, Charge all you can get, is no longer applicable. We are not allowed to consult with the man who asks such service, for he is not his own master any longer. It is a settled principle of law that agreements or promises made under duress or compulsion are not binding. Every day that passes sees some notary or justice going through the form of examining a married woman "apart from her husband," to ascertain that she signs a deed freely without any compulsion. If a man promise a burglar or a robber or a foot-pad who puts a pistol to his head, to pay to-morrow one hundred dollars, as a ransom for his life and property, the promise is not held to be binding. It was extorted.

Every day that passes sees men and women suffering, some in one way and some in another, yet all of them suffering so keenly that they are willing to pay anything that you choose to charge for relief. In such cases, what *shall* a man charge?

A man owns a lot and house. The city needs a street. What shall he charge for the right of way? If the man be a generous, public spirited man, he will be apt to wrong himself in view of the great benefit to the people. If he be avaricious, he will be apt to wrong the public. Hundreds of men there be who do not think themselves avaricious, who nevertheless feel free to take money from the public treasury, all that they can get.

Again: Men who are exceptionally endowed; what shall they charge for services that they are able to render almost without cost? What shall Jenny Lind or Madam Nilsson charge for a song? Jenny Lind [Goldsmith] in her prime sang cradle songs to her baby for nothing. Shall she sing to the rest of mankind for cash only? True, the rest of mankind have no claim on her. They have no right to her melody. Her song power is the free gift of God. What use shall she make of it? "Freely ye have received, freely give," has a beautiful sound. Is it worth anything as a practical guide?

The gifts that make a surgeon are native gifts rather than acquirements. What shall Brown Sequard charge for a five-minute consultation and a three-minute operation or prescription or opinion? No man has any right to extort service from his fellow man. But there is another question: Has any man a right to withhold service from his fellow man, when, by the good gift of God, he has been specially endowed to that service?

Or again: In the extreme complexity of our civil laws and commercial usages it by and by appears that only a few men in a great city like New York are so endowed that they are able to thread the labyrinth with certainty and see through to judgment. What shall they charge for their rare but easy skill? It is not a labor to them. It is an inspiration.

What shall a minister charge for sermons that it is easier to preach than to hold in?

Dare any man reply to his own conscience: "Charge all that you can get."

In short, are there not relations between man and man of such wide inequality that in their measure the same law applies to them that applies to the relation of all mankind to God? Romance, sentiment, and rhapsody wholly to one side—to the eye of ordinary reason and justice, is it not clear that man, self-compelled, urged on by his own greatness and nobleness, must needs be a law to himself and imitate in his deeds of grace the rain and the sunshine which all men need, but no man can pay for?

In addition to the mechanical doctrines of political economy, have we not need, in a commercial society, of something besides? What shall a mother charge a child for a mother's care? What shall one man charge another man for service when the inequality between them is as great as between mother and child? The little child and the little man are helpless. They are in the power of the great. But what shall the great charge?

Between the free and the equal, if such can be found, the rule holds true: Charge what you can collect. Between unequals the rule rarely applies. Oppressions, cruelties, hatreds, must needs come to pass if men in the adjustment of their acts one to another can find and obey no rules higher than those that are writ by political economists.

